# Does patellar resurfacing matter in robotic‐assisted total knee arthroplasty with functional alignment principles?

**DOI:** 10.1002/ksa.12769

**Published:** 2025-07-07

**Authors:** Emanuele Diquattro, Pietro Gregori, Christos Koutserimpas, Vasileios Giovanoulis, Elvire Servien, Cécile Batailler, Sébastien Lustig

**Affiliations:** ^1^ Orthopaedics Surgery and Sports Medicine Department, FIFA Medical Center of Excellence, Croix‐Rousse Hospital Lyon University Hospital, Hospices Civils de Lyon Lyon France; ^2^ SC Ortopedia‐Traumatologia e Chirurgia Protesica e dei Reimpianti di Anca e Ginocchio, IRCCS Istituto Ortopedico Rizzoli Bologna Italy; ^3^ Fondazione Policlinico Universitario Campus Bio‐Medico Roma Italy; ^4^ School of Rehabilitation Health Sciences University of Patras Patras Greece; ^5^ LIBM‐EA 7424, Interuniversity Laboratory of Biology of Mobility Claude Bernard Lyon 1 University Lyon France; ^6^ Univ Lyon, Claude Bernard Lyon 1 University, IFSTTAR, LBMC UMR_T9406 Lyon France

**Keywords:** anterior compartment, functional alignment, functional outcomes, image‐based robotic‐assisted system, patellar resurfacing, total knee arthroplasty

## Abstract

**Purpose:**

While new philosophies in total knee arthroplasty (TKA) aim to optimize alignment and ligament balancing, restoring the anterior compartment and understanding the consequences of patella resurfacing remain challenging. This study evaluates the functional consequences of anterior compartment restoration, with a specific focus on patella resurfacing when performing robotic‐assisted TKA using functional alignment (FA) principles.

**Methods:**

This retrospective comparative study included 268 patients affected by an end‐stage varus osteoarthritis who underwent robotic TKA according to FA principles. Patients were divided into two main groups based on whether patellar resurfacing procedure was performed (study group) or not (control group). Restoration of the anterior compartment and functional outcomes were evaluated using radiographic assessments, along with the Knee Society Score (KSS Knee and KSS Function), Forgotten Joint Score (FJS) and Kujala Score, all recorded at a minimum follow‐up of 2 years.

**Results:**

One hundred thirty‐eight cases underwent patellar resurfacing and 130 did not. Patellar tilt and patellar translation were reduced by 74.63% (*p* < 0.005) and 69.89% (*p* < 0.005) in the resurfaced group compared to the non‐resurfaced group, respectively. The patellar offset values showed no significant differences between the two groups (*p* = 0.873), although both groups showed a significant reduction in patellar offset in the post‐operative compared to the preoperative, 10.31% and 14.29%, respectively, in the resurfaced and non‐resurfaced groups (*p* < 0.05). KSS knee and functional scores were higher in the non‐resurfaced group compared to the resurfaced group (*p* = 0.023 and *p* = 0.017, respectively). Kujala and FJS scores were similar between the two groups.

**Conclusion:**

Both resurfaced and non‐resurfaced approaches improve radiographic outcomes regarding the anterior knee compartment after TKA performed with FA and image‐based robotic system, without signs of superiority in clinical outcomes in the resurfaced group. The decision of patella resurfacing can be made on an individualized basis.

**Level of Evidence:**

Level II.

**Abbreviations**
FA: functional alignment
FJS‐12: Forgotten Joint Score‐12
IQR: interquartile range
KA: kinematic alignment
KSS: Knee Society Score
MA: mechanical alignment
PO: patellar offset
PTi: patellar tilt
PTr: patellar translation
ROM: Range Of Motion
rTKA: robotic‐assisted TKA
TKA: total knee arthroplasty

## INTRODUCTION

The decision to resurface the patella in total knee arthroplasty (TKA) remains a topic of ongoing debate. The choice must be made on a case‐by‐case basis, considering the patient's symptoms, the presence of patellar osteoarthritis and the specific design of the implant [10]. This consequently has a great impact on the femoral biomechanics and the anterior compartment after a TKA [8].

With robotic assistance and personalized surgical strategies, interest in the anterior patellofemoral compartment has increased [2, 14, 17, 21]. On the other hand, the conventional jig instrumentation has limitations when it comes to restoring the anterior compartment [5, 9]. Minor defects in the anatomical restoration of this compartment can potentially lead to post‐operative complications such as patellar maltracking, severe pain and poor functional results [26, 33]. Patellar tracking is dictated by surgical techniques, implant placement, alignment used and implant characteristics in terms of femoral and patellar component design [4, 12, 16, 42, 44]. Although most modern implants aim to replicate the native shape of the trochlea, even today these trochlear and patellar prosthetic designs differ from the native ones, so surgical technique factors still remain crucial in attempting to achieve optimal tracking, reduced pain and post‐operative complication rates [24, 34, 35].

The robotic approach allows for a strict evaluation of the patient's specific anatomy by integrating the preoperative computed tomography (CT), with which the trochlear shape and patellar tracking can be displayed during the planning, as well as the procedure [1, 11, 45]. This allows the surgeon to evaluate the patellar tracking in real time, as well as the offset (under‐or‐over‐stuffing) of the anterior compartment space, and thus to make further modifications [39].

Although previous studies have addressed patellar resurfacing, limited data exist on its impact in robotic‐assisted TKA under functional alignment (FA) principles. This comparative study between resurfaced and non‐resurfaced groups was to evaluate the following outcomes: (1) the patellar offset (PO), (2) patellar tilt, (3) lateral patellar translation, and finally, the (4) clinical outcomes at least two years of follow‐up. The hypothesis was that achieving better radiograph values of these patellar parameters in the anterior compartment, performing patellar resurfacing, would have an impact on functional results after the robotic TKA in FA alignment principles.

## METHODS

This retrospective single‐centre comparative study included 268 consecutive patients who underwent primary image‐based robotic‐assisted TKA (MAKO, Stryker Corp.) for end‐stage varus osteoarthritis between March 2021 and January 2023, utilizing a prospectively maintained database, with a 2‐year minimum follow‐up. The exclusion criteria were revision cases, previous femoral osteotomy, post‐traumatic femoral osteoarthritis and preoperative valgus alignment on weight‐bearing X‐rays [hip–knee–ankle angle [HKA] > 180°] due to potential bias in patellofemoral tracking.

**Figure 1:**
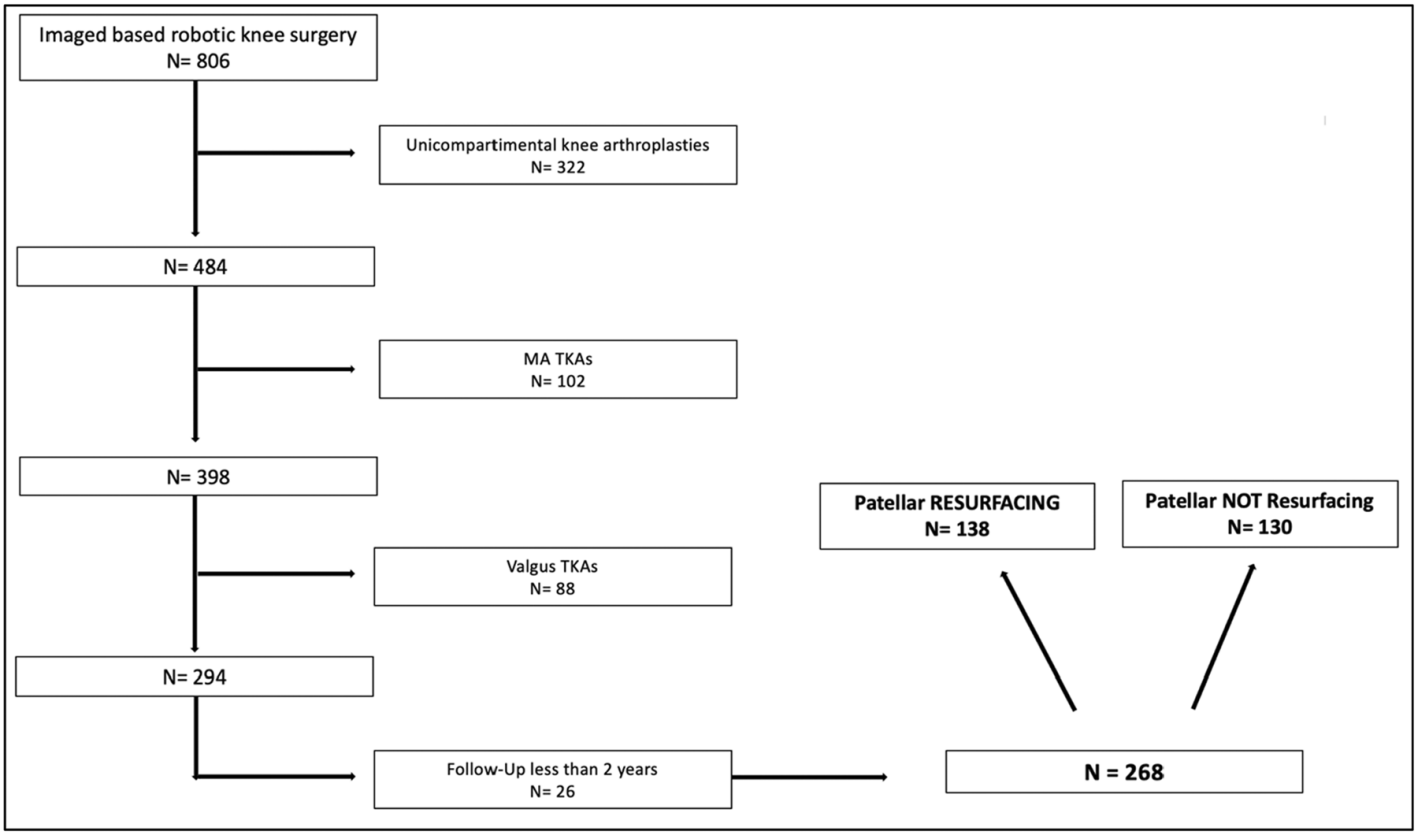
Detailed flowchart of enrolment in the study. Period: March 2021 and January 2023. MA, mechanical alignment; N, number of cases; TKA, total knee arthroplasty.

Patients undergoing robotic knee unicompartmental arthroplasty or robotic TKA with mechanical alignment philosophy due to soft tissue envelope insufficiency were excluded from the study. Two hundred ninety‐four primary TKAs performed by the image‐based robotic‐assisted system applying the FA principles [38] were included. Twenty‐six cases were lost during the follow‐up (Figure 1).

The medial subvastus approach was used in all cases. A tourniquet was not applied. The implant was the same for all the patients, using two different types of inserts: a posterior‐stabilized (PS) TKA design (Triathlon PS, Stryker Corp.) was used in 191 cases (64.97%); a cruciate substitution TKA design (Triathlon PS, Stryker Corp.) was used in the other 103 cases (35.03%). Both inserts have shown similar outcomes in functionally aligned robotic TKAs [20]. MAKO Total Knee Planning software (Stryker) built customized three‐dimensional knee models based on preoperative CT scans. The implant was positioned on this model based on the FA principles. Accuracy within 1 mm between the implant planning and final position is already proven [41]. The patella was resurfaced in 138 cases (study group). The criteria for resurfacing were anterior knee pain, patellofemoral osteoarthritis (Iwano ≥ 2), inflammatory arthritis or crystalline arthropathy. Patella was also resurfaced in cases with excessive patellar tilt or intraoperative maltracking [1, 26]. In 130 cases (control group), this procedure was not performed (Figure 1). The patellar resurfacing procedure was performed in a standardized manner by the participating surgeons using the manual technique (as this step is not supported by the robotic system's cutting guide). The patellar component was medialized as much as possible to avoid overhang, with concomitant lateral facetectomy when needed to address impingement, maltracking with lateralization tendency, and excessive external rotation against the femoral component [1, 26].

CT scan and X‐ray series, including anteroposterior, lateral in 30° flexion, weight‐bearing long‐leg film and skyline view, were done preoperatively for every patient. All radiographic measurements were made using PACS software (Centricity™, GE Healthcare). The radiographic assessment of the PO, together with the patellar translation and tilt, has been calculated to examine how the radiographic parameters varied in the anterior compartment [19]. The PO preop and postop [28], the patellar translation post‐op [43] and the patellar tilt post‐op [13] were measured using standardized and documented techniques. Knee Society Score (KSS) (knee and function scores) [7] were collected preoperatively and at a minimum follow‐up of 2 years. The Forgotten Joint Score (FJS) [19] and Kujala score [15] were collected at the final follow‐up.

All measurements were performed using the MAKO Robotic Platform Planning software (Mako, Stryker Corp). A calibrated scale in millimetres allowed accurate and reliable measurements, with an accuracy of 1 mm. The radiologic evaluations of the anterior compartment after TKA were performed by two independent orthopaedic surgeons (E.D. and P.G.).

### Statistical analysis

Distribution was evaluated with the Kolmogorov–Smirnov test. Independent *t*‐test or Mann–Whitney *U* test was used to compare groups based on the presence or absence of normality in the distribution, respectively. The Wilcoxon test was used to compare the desired parameters within the same population. Significance was set to *p* < 0.05. Sample size calculation was performed with G*Power 3.1. The minimum number of patients per group is approximately 64 to achieve a power of 80% with an effect size of 0.5, a significance level of 0.05 and using a two‐tailed Student's *t*‐test.

### Ethical approval

All procedures were performed in accordance with the ethical standards of the institutional and/or national research committee, the 1964 Helsinki Declaration and its later amendments, or comparable ethical standards. Data collection and analysis were carried out in accordance with MR004 Reference Methodology from the Commission Nationale de l'Informatique et des Libertés (Ref. 2229975V0). As per institutional standards, formal patient consent is not required for this type of study.

## RESULTS

A total of 268 patients (171 females, 63.81%) were enroled in this study, with a median age of 70 years, and a median body mass index = 28.125 kg/m^2^ (interquartile range [IQR]: 6–58). The mean follow‐up of the total population was 2.67 ± 0.53 years (IQR: 26).

### Radiographic evaluation

Radiographic evaluations showed significant differences in PO between pre‐ and post‐operative values in both groups (*p* < 0.05, Wilcoxon test), reducing by 10.31% in the resurfaced (mean post‐operative value 2.677 cm vs. mean preoperative value 2.985 cm) and 14.29% in the non‐resurfaced group (mean post‐operative value 2.679 cm vs. mean preoperative value 3.127 cm). No significant differences were shown in post‐operative offset values between the two groups (*p* = 0.873, Mann–Withney *U*‐test) (Graphics 1, 2, 3).

**Graphic 1:**
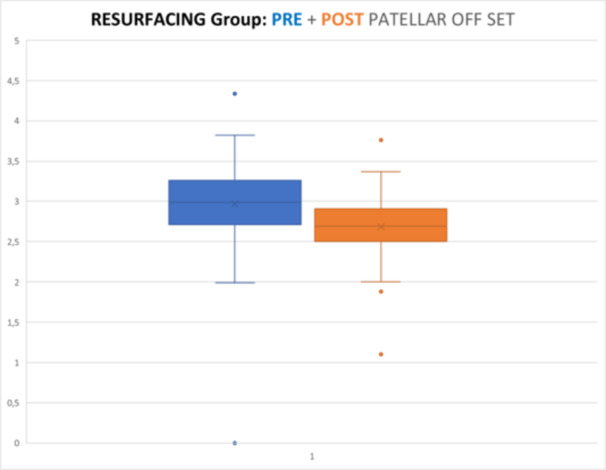
Resurfacing group (study group): preop versus postop patellar offset (*p* < 0.005).

**Graphic 2:**
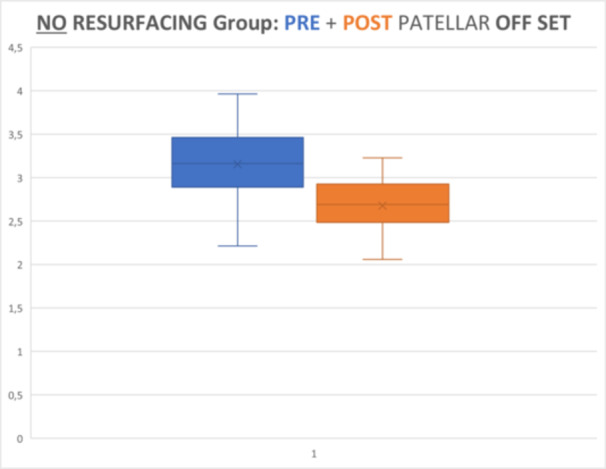
Not resurfacing group (control group): preop versus postop patellar offset (*p* < 0.005).

**Graphic 3:**
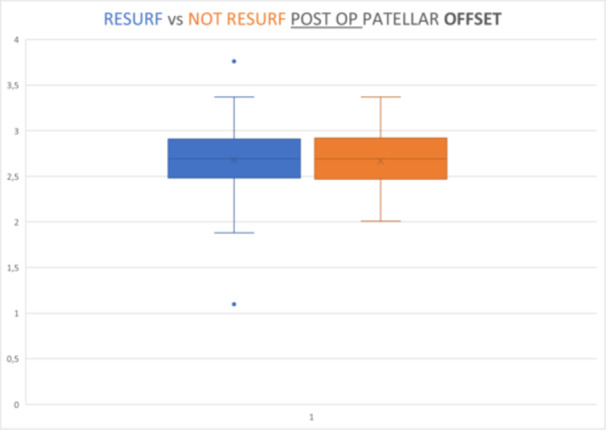
Resurfacing versus not resursufacing postop patellar offset (*p* = 0.873).

Patellar translation was less lateralized after patellar resurfacing compared to those that were not resurfaced. The post‐operative values of patellar translation showed a reduction by 69.89% in the resurfaced group compared to the non‐resurfaced group (mean value −0.141 cm in the resurfaced vs. −0.468 cm in the non‐resurfaced group, *p* < 0.05, Student's *t* test) (Graphic 4).

**Graphic 4:**
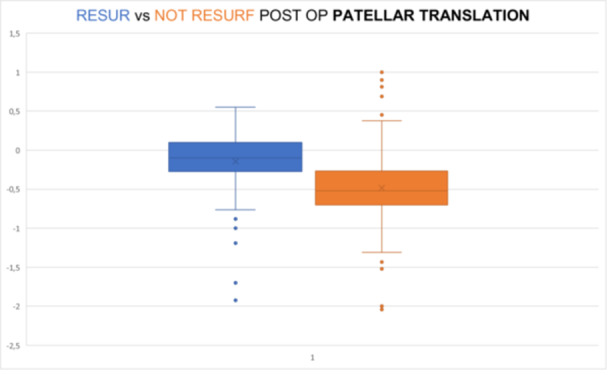
Resurfacing versus not resurfacing postop patellar translation (*p* < 0.005).

Finally, the differences between the post‐operative values of the patellar tilt value between the resurfaced group and the non‐resurfaced group was also found to be significant (*p* < 0.05, Student's *t*‐test), reduced by 74.63% in the non‐resurfaced group (mean value 1.498° in the resurfaced group vs. 5.905° in the non‐resurfaced group) (Graphic 5).

**Graphic 5:**
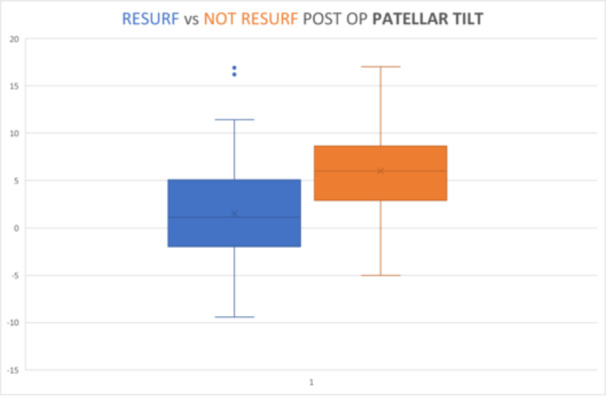
Resurfacing versus not resurfacing postop patellar tilt (*p* < 0.005).

The *p*‐Value < 0.005 indicates a highly significant difference in patellar tilt and translation between the groups, suggesting clinical interest.

### Clinical outcomes

There was no statistically significant difference in the preoperative KSS‐knee and KSS‐function scores (*p* = 0.21 and *p* = 0.044, respectively). The KSS‐knee and the KSS‐function post‐operative scores were significantly lower in the resurfaced group (*p* = 0.023 and *p* = 0.017, respectively). The differentials of the respective KSS knee and KSS function scores were also significantly lower in the resurfaced group (*p* = 0.027 and *p* = 0.046, respectively). KSS knee and function improved by an average of 3.69 points (*p* < 0.05) and of 4.27 points (*p* < 0.05), respectively, in the non‐resurfaced group.

No statistically significant differences were found in the post‐op Kujala (*p* = 0.72) and in FJS as well (*p* = 0.71) (Tables 1, 2, 3).

**Table 1:** Not patellar resurfacing group (control group) scores.

Scores	Preoperative	Postoperative	Difference post–pre
KSS Knee score (mean ± SD) [min; max]	63.25 ± 13.15 [29; 94]	93.22 ± 8.93 [47; 100]	14.79 ± 19.22
KSS Function score (mean ± SD) [min; max]	66.03 ± 16.88 [5; 100]	93.14 ± 8.78 [49; 100]	13.41 ± 19.5
FJS score (mean ± SD) [min; max]	NA	75.61 ± 22.2 [0; 100]	NA
Kujala score (mean ± SD) [min; max]	NA	87.52 ± 15.79 [27; 100]	NA

Abbreviations: FJS, Forgotten Joint Score; KSS, Knee Society Score; SD, standard deviation.

**Table 2:** Patellar resurfacing group (study group) scores.

Scores	Preoperative	Postoperative	Difference post–pre
KSS Knee score (mean ± SD) [min; max]	65.45 ± 13.14 [16; 94]	91.73 ± 8.14 [58; 100]	13.54 ± 17.71
KSS Function score (mean ± SD) [min; max]	67.78 ± 15.7 [28; 100]	90.61 ± 9.92 [58; 100]	11.81 ± 17.48
FJS score (mean ± SD) [min; max]	NA	76.03 ± 22.95 [0; 100]	NA
Kujala score (mean ± SD) [min; max]	NA	89.5 ± 12.54 [32; 100]	NA

Abbreviations: FJS, Forgotten Joint Score; KSS, Knee Society Score; SD, standard deviation.

**Table 3:** Statistical analysis.

Scores	Resurfaced	Nonresurfaced	*p*‐**Value**
Preop KSS‐knee	65 (IQR 57–77)	65 (IQR 54–73)	0.21
Preop KSS‐function	70 (IQR 60–80)	70 (IQR 55–80)	0.44
Post‐op KSS‐knee	90 (IQR 90–100)	95 (IQR 90–100)	0.023
Post‐op KSS‐function	90 (IQR 88–100)	95 (IQR 90–100)	0.017
Difference in KSS knee	26 (IQR 17–36.25)	30 (IQR 20–41.75)	0.027
Difference in KSS function	20 (IQR 10–35)	26 (IQR 17.25–40)	0.046
Post‐op Kujala	92 (IQR 84–100)	92 (IQR 82–100)	0.72
Post‐op FJS	82 (IQR 63–92)	82 (IQR 63–92)	0.71

*Note*: Data rejected normality (Kolmogorov–Smirnov test). Presented as median (IQR). Comparisons were made with the Mann–Whitney *U* test.

Abbreviations: FJS, Forgotten Joint Score; IQR, interquartile range; KSS, Knee Society Score.

## DISCUSSION

The main findings of this study were that in robotic TKA performed using FA principles, patellar resurfacing was associated with improved radiographic restoration of anterior compartment parameters; however, no significant differences in clinical outcomes were observed between the resurfaced and non‐resurfaced groups at a minimum follow‐up of 2 years.

Current literature lacks robust evidence regarding the impact of patellar parameters on outcomes in robotic‐assisted TKA. With traditional instrumentation for MA, the tibiofemoral joint is often prioritized, while the complexity of the patellofemoral joint may be overlooked due to a primary focus on femoral component rotation and lateralization. This limited consideration of the anterior compartment may result in early complications or revision surgeries due to patellofemoral issues [22].

In this study, we evaluated radiographic patellar parameters such as patellar tilt and patellar translation, which were found to be significantly lower in patients who underwent patellar resurfacing compared to those who did not. In cases where it is indicated, patellar resurfacing is capable of restoring these radiographic parameters more accurately than preserving the native arthritic patella [18]. These radiographic values, when outside the optimal range, can influence clinical outcomes, as other studies have already demonstrated [29].

How the anterior compartment in terms of offset may vary has already been discussed in a previous clinical study, which showed a tendency to understuffing the anterior compartment after primary TKA performed with a robot‐assisted system based on imaging and FA principles [17]. The trochlear offset was mostly understuffed after TKA compared to the native anatomy, mainly for medial and lateral condyles at 30° and 70° of flexion. The global anterior compartment restoration was understuffed in full extension (−0.7 ± 2 mm), at 30° (−4.4 ± 2 mm) and 70° of flexion (−3.6 ± 2.5 mm). At 90°, the global anterior compartment restoration was overstuffed (2.2 ± 1.8 mm). Furthermore, a significant impact on functional outcomes (KSS functional score and knee flexion) was found when the anterior compartment was overstuffed at 70° and 90° [17].

Although not statistically significant, reducing patellar offset reduces the risks of flexion stiffness and anterior pain after TKA, clinical signs that often correlate with radiographic changes such as increased lateral patellar translation and increased patellar tilt [3, 27]. None of our cases suffered complications related to offset reduction or understuffing of the anterior compartment (maltracking, patellar luxations, mobilization of patellar prosthetic components).

Despite some meta‐analyses reporting better functional outcomes in cases of TKA with resurfacing [29], this discordance in evaluated clinical scores may not be directly correlated with the radiographic parameters obtained [28, 31]. A recent meta‐analysis showed that patellar resurfacing in combination with a modern patellar‐friendly implant was not associated with a lower rate of anterior knee pain, complications or re‐interventions than non‐prosthetic resurfacing, nor did it provide a clinically significant improvement in specific knee function. In contrast, patellar resurfacing in combination with an ‘unfriendly’ TKA implant was associated with a significantly better OKS and a lower re‐intervention rate. Implant design must be considered when patellar resurfacing is taken as an option [40]. Optimization of radiographic patellar references restoration with TKA should be further explored in the future development of modern TKA.

Additionally, current prosthetic designs, which fail to restore the anterior femoral anatomy, combined with MA methods, contribute to a mismatch between the native and prosthetic patellofemoral compartments [35]. Specifically, the trochlear design was created for MA, featuring a trochlear axis with a 6° angle relative to the distal condylar axis and dysplastic morphologies [36, 37]. Using preoperative CT scans, robotic‐assisted systems based on imaging can accurately match the natural shape of the trochlea during the planning stage. The femoral implant is overlaid on the CT scan and carefully adjusted to match the native trochlea's position. These customized alignment techniques, facilitated by image‐guided robotic systems, have demonstrated superior precision in assessing and restoring the trochlear groove compared to traditional MA or KA approaches [25, 32]. MA resulted in the greatest deviation of the trochlear groove from its natural anatomy compared to KA and FA. KA led to unsafe coronal implant positioning in at least 13% of cases and caused internal rotation of the femoral component beyond 3° in over 25% of cases. In contrast, FA exceeded coronal safety limits in only 3.2% of patients and rotational safety limits in just 1.7%. However, these personalized surgical techniques tend to underfill the anterior compartment, as demonstrated in an in vitro study using an image‐based robotic system [38, 44].

In this cohort, the subvastus approach was utilized, providing adequate patellar stability without affecting the extensor mechanism, even in cases of moderate understuffing. Furthermore, the implementation of an image‐based robotic‐assisted system allowed for the accurate restoration of trochlear groove orientation and femoral rotation [38], resulting in improved intraoperative patellar tracking [6, 23, 30].

Several limitations should be acknowledged in this study. First, the average follow‐up period was relatively short. However, a 2‐year follow‐up is abundantly sufficient to evaluate post‐operative anterior pain and functional outcomes after primary TKA. This cohort will continue to be monitored over the coming years to assess long‐term functional progression. Second, the study was retrospective and involved a relatively small cohort. Nonetheless, the clinical data analyzed were systematically and prospectively recorded in medical files. A post‐hoc analysis confirmed satisfactory study power. Third, only one TKA design was evaluated. Since trochlear shape varies significantly across implants, focusing on a single design ensured consistency within the study. Additionally, the global anterior compartment restoration formula was derived by combining all patellar measurements. While this approach may be somewhat simplistic, it facilitates easier comparison and analysis.

## CONCLUSION

Both resurfacing and non‐resurfacing strategies improve radiographic outcomes regarding the anterior knee compartment after TKA performed with functional positioning and image‐assisted robotic system, without any difference in clinical outcome improvement in the resurfaced group. The decision of patella resurfacing can be made on an individualized basis and should not be systematic. Considering the benefit of patellar resurfacing for optimization of anterior compartment restoration with TKA, it should be further explored in the future development of modern TKA, especially with the accuracy of robotic systems.

## AUTHOR CONTRIBUTIONS

**Emanuele Diquattro**: Conceptualization; methodology; data curation; writing—original draft. **Pietro Gregori**: Conceptualization; methodology; data curation; writing—original draft. **Christos Koutserimpas**: Data curation; methodology; writing—reviewing and editing. **Vasileios Giovanoulis**: Data curation; methodology; writing—reviewing and editing. **Elvire Servien**: Conceptualization; methodology; writing—reviewing and editing. **Cécile Batailler**: Conceptualization; supervision; validation; writing—reviewing and editing. **Sèbastien Lustig:** Conceptualization; supervision; validation; writing—reviewing and editing.

## CONFLICTS OF INTEREST STATEMENT

Elvire Servien: Consultant for Corin. Institutional research support from Corin, Amplitude. Cécile Batailler: Consultant for Stryker, Smith Nephew and Groupe Lepine. Sébastien Lustig: Royalties from Smith Nephew, Stryker and Serf. Consultant for Stryker, Heraeus; Institutional research support from Amplitude and Groupe Lepine; Editorial Board for Journal of Bone and Joint Surgery (Am). The remaining authors declare no conflicts of interest.

## ETHICS STATEMENT

The study has been approved by the scientific committee of Hospices Civils de Lyon, France. It adhered to the MR004 Reference Methodology of the French Commission Nationale de l'Informatique et des Libertés (Ref. 2229975V0). All patients provided written informed consent.

## Data Availability

The authors have nothing to report.
